# Enhanced short-wavelength sensitivity in the blue-tongued skink *Tiliqua rugosa*

**DOI:** 10.1242/jeb.244317

**Published:** 2022-06-13

**Authors:** Nicolas Nagloo, Jessica K. Mountford, Ben J. Gundry, Nathan S. Hart, Wayne I. L. Davies, Shaun P. Collin, Jan M. Hemmi

**Affiliations:** 1 School of Biological Sciences, The University of Western Australia, Crawley, WA 6009, Australia; 2 Department of Biology, Lund University, S-212263 Lund, Sweden; 3 The UWA Oceans Institute, The University of Western Australia, Nedlands, WA 6009, Australia; 4 Oceans Graduate School, The University of Western Australia, Crawley, WA 6009, Australia; 5 Clinical Genetics and Epidemiology, and Centre for Ophthalmology and Visual Science incorporating the Lions Eye Institute, The University of Western Australia, Nedlands, WA 6009, Australia; 6 School of Natural Sciences, Macquarie University, Sydney, NSW 2109, Australia; 7 Umeå Centre for Molecular Medicine (UCMM), Umeå University, S-90187 Umeå, Sweden; 8 School of Agriculture, Biomedicine and Environment, La Trobe University, Bundoora, VIC 3086, Australia

**Keywords:** Keyword: Spectral sensitivity, Electroretinography, Photoreceptors, Opsins, Scincidae

## Abstract

Despite lizards using a wide range of colour signals, the limited variation in photoreceptor spectral sensitivities across lizards suggests only weak selection for species-specific, spectral tuning of photoreceptors. Some species, however, have enhanced short-wavelength sensitivity, which probably helps with the detection of signals rich in ultraviolet and short wavelengths. In this study, we examined the visual system of *Tiliqua rugosa*, which has an ultraviolet/blue tongue, to gain insight into this species' visual ecology. We used electroretinograms, opsin sequencing and immunohistochemical labelling to characterize whole-eye spectral sensitivity and the elements that shape it. Our findings reveal that *T. rugosa* expresses all five opsins typically found in lizards (SWS1, SWS2, RH1, RH2 and LWS) but possesses greatly enhanced short-wavelength sensitivity compared with other diurnal lizards. This enhanced short-wavelength sensitivity is characterized by a broadening of the spectral sensitivity curve of the eye towards shorter wavelengths while the peak sensitivity of the eye at longer wavelengths (560 nm) remains similar to that of other diurnal lizards. While an increased abundance of SWS1 photoreceptors is thought to mediate elevated ultraviolet sensitivity in a couple of other lizard species, SWS1 photoreceptor abundance remains low in this species. Instead, our findings suggest that short-wavelength sensitivity is driven by multiple factors which include a potentially red-shifted SWS1 photoreceptor and the absence of short-wavelength-absorbing oil droplets. Examining the coincidence of enhanced short-wavelength sensitivity with blue tongues among lizards of this genus will provide further insight into the co-evolution of conspecific signals and whole-eye spectral sensitivity.

## Abstract (precis)

**Highlighted Article:** Multiple facets of the visual system have adapted to provide enhanced sensitivity to short-wavelength light in the blue-tongued skink. This opens the door to studying how the blue tongue and the visual system which perceives it have co-evolved.

## INTRODUCTION

There are approximately 8000 reptilian species, inhabiting a range of aquatic and terrestrial environments (Hickman et al., 2008). Among them, lizards are the most ecologically diverse group with representatives occupying a range of terrestrial, fossorial, aquatic, arboreal and aerial habitats (Hickman et al., 2008). In many species examined thus far, adaptive radiation of the visual system appears to mirror the ecological diversity of lizards (Walls, 1942), where vision plays a crucial role in predation and conspecific communication (Kirmse et al., 1994; Stapley and Whiting, 2006). However, previous studies have mainly focused on well-known subgroups such as geckoes, iguanids and chameleons (Crescitelli, 1977; Govardovskii et al., 1984; Loew et al., 2002). As such, there is a clear gap in our understanding of visual ecology for many other common and ecologically important subgroups of reptiles.

During important intra-specific interactions, such as courtship displays and male-to-male fitness signalling, lizards use a variety of colourful conspecific signals which often extend beyond the visible spectrum and into the ultraviolet. This has led visual ecologists to investigate whether lizard visual systems are more sensitive to specific wavelengths that would facilitate the detection of these coloured signals (Fleishman et al., 1997; Loew et al., 2002). Previous experiments have used microspectrophotometry (MSP) to obtain the absorbance spectra of specific photopigments, photoreceptor types and oil droplets (Barbour et al., 2002; Govardovskii et al., 1984; Katti et al., 2019; Kawamura and Yokoyama, 1998; Takenaka and Yokoyama, 2007) or electroretinograms (ERGs) to measure the spectral sensitivity of the whole eye (Arden and Tansley, 1962; Ellingson et al., 1995; Fleishman et al., 1997; Forbes et al., 1960; Hamasaki, 1968).

Data generated using MSP from 17 species of Caribbean anole lizards revealed the presence of four spectrally distinct photoreceptor types with little variation in peak sensitivity across species, despite the great variety of dewlap colours involved in conspecific signalling. The wavelengths of peak sensitivity (λ_max_) for the four photoreceptor types are 365 nm (ultraviolet-sensitive, UVS), 456 nm (short-wavelength-sensitive, SWS), 494 nm (middle-wavelength-sensitive, MWS) and 564 nm (long-wavelength-sensitive, LWS) (Loew et al., 2002). The λ_max_ of these photoreceptor types seem to also be broadly conserved in geckoes, iguanids, skinks and lacertids (Martin et al., 2015) with only small deviations. The four spectrally distinct photoreceptor types typically found in diurnal lizards are generally the result of the expression of four cone opsin genes, namely a long-wavelength-sensitive (*LWS*) opsin gene, a rhodopsin-like 2 (*RH2*) opsin gene, short-wavelength-sensitive 2 (*SWS2*) and short-wavelength-sensitive 1 (*SWS1*) opsin genes, and a single rod (*RH1*) opsin gene (Katti et al., 2019; Kawamura and Yokoyama, 1997; Martin et al., 2015; Yewers et al., 2015). Unlike most vertebrates that possess rod photoreceptors that express *RH1*, the rod opsin gene in some species of lizards is expressed in single, cone-like photoreceptors, and this is widespread across diurnal lizards, e.g. *Chamaeleo chamaeleon*, *Anolis carolinensis* and *Tiliqua rugosa* (Bennis et al., 2005; McDevitt et al., 1993; New et al., 2012). At the amino acid level, visual opsins show high sequence identity within each opsin class across lizard species. These opsins are bound to a retinal-based chromophore which can shift the spectral peak and broaden the bandwidth of the individual photopigment class. While most terrestrial vertebrates, including diurnal lizards, are known to use an A_1_-based chromophore (i.e. 11-*cis* retinal), mixtures of both A_1_ and A_2_ (3,4-didehydroretinal) chromophores have been observed in *Podarcis sicula*, *Chameleo dilepis* and *Fucifer pardalis* (Bowmaker et al., 2005; Provencio et al., 1992). Pure A_2_ chromophore is found only in *A. carolinensis* and *Zootoca vivipara* (Kawamura and Yokoyama, 1993; Loew et al., 2002; Martin et al., 2015). The combination of A_1_ and/or A_2_ chromophores bound to visual opsins is therefore an important mechanism of adaptive spectral tuning (Corbo, 2021; Hárosi, 1994; Whitmore and Bowmaker, 1989).

ERGs of anole lizards (*Anolis gundlachi*, *Anolis cristatellus*, *Anolis krugi*, *Anolis pulchellus*, *Anolis stratulus*, *Anolis evermanni* and *Anolis sagrei*), horned toads (*Phrynosoma* spp*.*), spiny lizards (*Scleroporus* spp*.*) and geckoes (*Gonatodes albogularis*) suggest that the spectral sensitivity of the whole eye is as conserved as the spectral sensitivity of photoreceptor types (Arden and Tansley, 1962; Ellingson et al., 1995; Fleishman et al., 1997; Forbes et al., 1960). The typical spectral sensitivity curve of the diurnal lizard eye is characterized by a broad shoulder of high sensitivity between 530 nm and 590 nm and a secondary (less sensitive) peak at approximately 360 nm (Fleishman et al., 1997). Fleishman et al. (1997) have hypothesized that the convergence of these spectral sensitivity curves may be driven by the common need to detect objects against green vegetation in the background, which has a peak reflectance at 550 nm (Fleishman et al., 1997). Despite this pattern of conserved spectral sensitivity, a more recent study has revealed enhanced sensitivity at 360 nm (ultraviolet) in the retina of the cordylid lizard, *Platysaurus broadleyi* (Fleishman et al., 2011), which correlates with an increased abundance of UVS receptors. While colour discrimination models have revealed that this enhanced sensitivity would facilitate the detection and discrimination of conspecific signals rich in ultraviolet, it remains difficult to determine whether the spectral properties of conspecific signals can shape the spectral sensitivity of diurnal lizards. Additional studies examining the spectral sensitivity of the whole eye across lizard taxa which make use of conspicuous visual signals are necessary to untangle the multiple factors that drive spectral sensitivity and signal detection in diurnal lizards.

Sleepy lizards, *Tiliqua rugosa*, are a species of blue-tongued lizard in which highly aggressive male-to-male interactions can lead to scarring and scale loss (Murray and Bull, 2004). Displays of their blue tongue are thought to play a role in male-to-male signals and could be used to avoid costly physical altercations during the mating season (Abramjan et al., 2015; Murray and Bull, 2004). Recent work in the common blue tongue lizard *Tiliqua scincoides* also suggests that the tongue might be a deterrent to predators by providing a sudden overwhelming flash of ultraviolet and blue light that would intimidate and startle predators (Badiane et al., 2018). These blue tongues are not limited to the *Tiliqua* genus but have also been observed in closely related large skinks with tongue colour ranging from pale grey to dark blue, with primary peak reflectance at ∼320 nm and a secondary peak at ∼460 nm (Abramjan et al., 2015). The presence of blue tongues across several genera and their conspicuous nature offers an opportunity to closely examine the phylogenetic and environmental factors that drive whole-eye spectral sensitivities in these lizards. Here, we propose to start with a thorough examination of the visual system of *T. rugosa* to build on prior knowledge of its retinal organization.

New et al. (2012), previously showed that the retina of the sleepy lizard contains only cones, with ∼20% of the cone population expressing the RH1 opsin. Both single and double cones were observed, with the presence of pale-yellow oil droplets reported in single cones and the principal member of the double cones (New et al., 2012). The density of cones and retinal ganglion cells peaks in the retinal centre with densities reaching 76,000 cells mm^−2^ and 15,500 cells mm^−2^, respectively (New and Bull, 2011; New et al., 2012). Anatomical estimates of the visual acuity suggest the sleepy lizards have a spatial resolving power of 6.8 cycles deg^−1^ (New and Bull, 2011). The current study used ERGs to measure the spectral sensitivity of the eye, opsin sequencing to characterize the full complement of opsin genes expressed, and immunohistochemistry and design-based stereology to visualize and map the complement of photoreceptors. The findings are discussed in relation to the visual ecology and behaviour of *T. rugosa* in comparison to other lizard species*.*

## MATERIALS AND METHODS

### Animals

Seven adult *Tiliqua rugosa* (Gray 1825) were caught around the Perth metropolitan area (WA, Australia) and housed on the campus of The University of Western Australia for less than 1 year. An ultraviolet-emitting light globe (Repti Glo UVB10 Compact 13W, Exo-Tera, Montreal, QC, Canada) provided animals with a full-spectrum light and a 12 h:12 h light:dark photoperiod. All captive lizards were mature adults with a body mass ranging from 450 to 670 g and were fed on a diet of soft vegetables supplemented with crickets and vitamins. All experimental procedures were approved by the ethics committee of The University of Western Australia (AEC no. RA/3/100/1030). All animals used in this study were euthanized according to protocols outlined in the ethics application above, with an intravenous injection of sodium pentobarbital.

### Transmission of the optical elements of the eye

The transmission of the lens and cornea in the eyes of *T. rugosa* was measured using frozen samples. While the transmission of the vitreous can be affected by freezing, the lens and cornea remain largely unchanged, especially at shorter wavelengths (Pérez i de Lanuza and Font, 2014). The lens and cornea were thawed and placed on a perforated plate underneath an inverted integrating sphere (FOIS-1, Ocean Optics). A 600 µm fibre (P600-2-UV-Vis, Ocean Optics) carrying light from a pulsed-xenon arc lamp (PX-2, Ocean Optics) was aligned to the centre of the sample against the bottom surface so that light transmitted through the sample entered directly into the integrating sphere. The light from the integrating sphere was collected by a second 600 µm fibre (P600-2-UV-Vis, Ocean Optics) and carried to a spectrophotometer (USB2000+, Ocean Optics) for spectral analysis using SpectraSuite software (Ocean Optics). At the beginning of experimental measurements, light and dark references were taken to calibrate the spectrophotometer to ambient light conditions.

The spectral transmittance (330–800 nm) of the different retinal cone photoreceptor oil droplets was measured using a single-beam wavelength-scanning microspectrophotometer as described previously (Hart, 2004). Briefly, small pieces of retina were dissected out of a fresh eyecup and mounted between coverslips in a solution of 8% dextran in 0.1 mol l^−1^ phosphate buffered saline (pH 7.2). Oil droplet transmittance was measured against a reference measurement made through a patch of nearby retinal tissue to control for absorbance of retinal tissue. While we were able to reliably record from two types of oil droplets, the process was inherently difficult as a result of the small size and fragility of reptilian oil droplets. We are unlikely to have missed additional oil droplet types because of a size bias, as colourless or pale oil droplets tend to be smaller than the more conspicuous green, orange, red or yellow oil droplets (Goldsmith et al., 1984).

### Measuring spectral sensitivity using ERGs

Animals were anaesthetized with a combination of ketamine (Ceva Ketamine Injection; dosage: 50 mg kg^−1^) and medetomidine (Ilium Medetomidine Injection; dosage: 165 µg kg^−1^) administered intramuscularly during all electrophysiological experiments. Proxymetacaine hydrochloride (Alcaine 0.5%, Alcon) was applied to the corneal surface to provide additional local anaesthesia. A platinum active electrode was placed at the corneal surface with conductive gel, while a silver/silver-chloride reference electrode was placed behind the head and the ground connected to the Faraday cage. A differential amplifier (DAM50, World Precision Instruments) combined with a National Instruments data acquisition board (USB-6353, National Instruments) were used to amplify and collect the compound neural responses of the eye.

Spectral sensitivity was measured between 650 and 350 nm at 20 nm intervals and then from 340 to 640 nm at 20 nm intervals following a similar protocol and equipment to those used in previous studies (Jessop et al., 2020; Ogawa et al., 2015). The data were then spliced into a single spectral sensitivity curve from 350 to 650 nm with interleaved measurements every 10 nm. The stimulus consisted of an on/off flickering light alternating between coloured and white light and separated by dark intervals of equal duration produced by chopping the light path with a motorized wheel (Jacobs et al., 1996). Time intervals between white and coloured flashes were used to calculate signal frequency, with standard measurements carried out at 10 Hz. Monochromatic light (15 nm full width at half-maximum transmission) was produced by an automated monochromator (Polychrome V, Till Photonics), while white light was produced by a Xenon arc lamp (HPX-2000, Ocean Optics). The two light paths were combined using mirrors and a beam splitter and gathered into a 1.1 mm quartz optic fibre (Till Photonics). The end of the optical fibre was attached to an ultraviolet-transmitting quartz lens, which was placed ∼1.5 cm away from the eye. The proximity of the output fibre to the eye ensured that the light diverged and diffusely stimulated the retina at the back of the eye with coloured and white light stimulating the same retinal region. Irradiance of the coloured and white lights was measured using a ILT1700 radiometer with a SED033 detector combined with a flat response filter and a diffuser to achieve a cosine response profile (International Light Technologies Inc.). White light output from the optical setup reached a maximum irradiance of 3.11×10^−3^ W cm^−2^. The white light intensity could be adjusted using neutral density (ND) filters ranging from 0.8 to 1.3 ND to allow for a stronger white light response when adaptation lights were used but was held constant throughout a full spectral measurement. The irradiance of the coloured lights ranged from 1.76×10^−3^ to 7.25×10^−3^ W cm^−2^ for wavelengths ranging from 350 to 650 nm and was dynamically adjusted to produce the same response amplitude as white light. At each 10 nm interval from 350 to 650 nm, sensitivity was measured as the reciprocal of the number of photons needed for the monochromatic light to produce an equivalent electrical response to that produced by white light (Neitz et al., 1989).

To analyse the contribution of specific photoreceptor subpopulations to the overall response of the eye, spectral sensitivity was measured under a range of conditions designed to alter the contribution of different photoreceptor populations to the recorded signal. To isolate spectrally distinct photoreceptor types, a second monochromatic light (adaptation light from a Polychrome V, Till Photonics) was superimposed on the stimulus to selectively reduce contrast to cell populations that are maximally sensitive to different regions of the spectrum. In addition, the flicker rate of the stimulus was adjusted from 3 to 30 Hz by controlling the speed of the chopper wheel. This was expected to bias contributions from slow or fast photoreceptor populations to the overall ERG signal (Neitz et al., 1989). At the beginning of each experiment, animals were dark adapted for 1 h and a standard spectral sensitivity curve recorded. After each use of a bright adaptation light, the animal was dark adapted for at least a further 30 min before the next recording.

### Statistical comparison of spectral sensitivities across temporal frequencies

For any given comparison, the ERG curves were normalized by linearly fitting them to the average of all curves being compared. The ratio of short- to long-wavelength (SW:LW) sensitivity was then calculated and used to compare ERG responses across temporal frequencies. In this case, the ratio was obtained by comparing the integral of the curve on either side of 530 nm. This is halfway between the peak sensitivity of MWS (494 nm) and LWS (564 nm) photoreceptors previously reported in anole lizards (Loew et al., 2002) and allows for the comparison of LWS photoreceptors with all other photoreceptor subtypes. Reciprocal transformation of ratio data was used to reduce skewness in raw data. A generalized linear mixed effect model was then used on the transformed data with temporal frequency as a fixed effect and with individual identity as a random effect. This was implemented using the *fitglme* function in Matlab 2021a (MathWorks) with the following model definition: fitglme(data, SWratio∼temporal_frequency+(1|animal_id)).

### Isolation and sequencing of opsin mRNA

Eyes used for opsin sequencing were dissected and preserved in RNAlater (Sigma-Aldrich) at 4°C immediately after euthanasia of the animal and following enucleation of the eye. Total RNA was extracted from homogenized retinal samples using TRIzol reagent with the PureLink RNA Mini Kit (Thermo Fisher Scientific), following the steps outlined by the manufacturer. cDNA was subsequently generated using 2 µg of total RNA and the miScript II Reverse Transcription (RT) Kit (Qiagen), according to the manufacturer's instructions. The *LWS*, *SWS1*, *SWS2*, *RH2* and *RH1* genes were PCR amplified according to Davies et al. (2009) from cDNA using degenerate primers listed in Table S1 and as follows: DIAPLMF1, DIAPLMF2, DIAPLMR1 and DIAPLMR2 for the amplification of the *LWS* gene; DIAPS1F1, DIAPS1F2, DIAPS1R1 and DIAPS1R2 for *SWS1*; DIAPS2F1, DIAPS2F2, DIAPS2R1 and DIAPS2R2 for *SWS2*; DIAPPR2F1, DIPAPR2F2, DIAPR2R1 and DIAPR1R2 for *RH2*; and DIAPPR1F1, DIPAPR1F2, DIAPR1R1 and DIAPR1R2 for *RH1* (Davies et al., 2009; Hart et al., 2016; Knott et al., 2013). To confirm that a full complement of visual opsin genes was detected, a series of PCR experiments using AOAS primers (Table S1) was also performed. All PCR protocols and conditions are outlined in Davies et al. (2009): briefly, a first-round PCR was carried out using 200 ng of template cDNA with My Taq DNA polymerase (Bioline Alexandria): initial denaturation at 95°C for 5 min; 40 cycles at 95°C for 30 s, 50°C for 1 min, 72°C for 1.5 min; and a final extension at 72°C for 10 min. Resulting PCR products were diluted 1:10 and used as template for second round PCR using conditions as per first-round, except for an annealing temperature of 55°C. PCR products were visualized using agarose gel electrophoresis and subsequently cloned into a pGEM-T easy cloning vector (Promega). Blue/white colonies were screened using standard techniques and a test digestion with endonuclease restriction enzyme EcoR1 was carried out to confirm insertion. Positive clones were sequenced in both directions using Sanger sequencing (AGRF).

### Sequence alignment and phylogenetic analyses

A codon-matched nucleotide sequence alignment of 85 agnathan (jawless) and gnathostome (jawed vertebrate) opsin coding regions, ranging from lampreys to mammals, was generated by ClustalW (Higgins et al., 1996) and manually manipulated to refine the accuracy of cross-species comparison. Specifically, the alignment incorporated the opsin sequences of five visual photopigments expressed in the retina of *T. rugosa* (GenBank accession numbers: ON637851–ON637855) compared to those species listed in the phylogenetic tree. All five opsin classes were included, with several vertebrate ancient (VA) opsin sequences used collectively as an outgroup given that this opsin type is a sister clade to all five visual photopigment classes. Phylogenetic analyses of 1000 replicates were conducted in MEGA11 (Tamura et al., 2021), with evolutionary histories being inferred by using the Maximum Likelihood method and General Time Reversible model (Nei and Kumar, 2000). The percentage of trees in which the associated taxa clustered together is shown next to the branches. Initial trees for the heuristic search were obtained by applying Neighbour-Joining and BioNJ algorithms (Saitou and Nei, 1987) to a matrix of pairwise distances estimated using the Maximum Composite Likelihood (MCL) approach (Tamura and Nei, 1993). The tree was drawn to scale, with branch lengths measured in the number of substitutions per site. A total of 903 positions was present in the final dataset, with all positions with <95% site coverage being eliminated. That is, <5% alignment gaps, missing data and ambiguous bases were allowed at any position.

### Histological preparations

After completion of electrophysiological recordings, animals were euthanized with an intracoelomic injection of sodium pentobarbital (Lethabarb, Virbac; dosage: 200 mg kg^−1^). The dorsal region of the eye was cauterized prior to enucleation to allow for easy orientation. Once removed, the eye was opened with a small incision at the limbus using a scalpel blade and a small cut was made in the dorsal retina to maintain orientation. The cornea, lens and vitreous were removed and the eyecup preserved in 4% paraformaldehyde (PFA) in 0.1 mol l^−1^ phosphate buffer (PB, pH 7.2–7.4) for both immunohistochemical analyses and assessing retinal topography. Eyes were fixed in PFA for 24 h, then stored in 0.1 mol l^−1^ PB plus 0.1% sodium azide. Radial cuts were made to relieve tension across the hemisphere of the eyecup and allow the retina to be flattened for wholemounts. The sclera and retinal pigment epithelium were removed to expose the retina.

### Sampling of photoreceptor subtypes

Once the retinae were immunohistochemically labelled (see Supplementary Materials and Methods), or simply mounted in glycerol, their outline was digitized using an X4.0 NA 0.17 objective lens, a motorized stage (MAC200; Ludl Electronics Products) and StereoInvestigator software (MBF Bioscience). The optical fractionator probe developed by West et al. (1991) and adapted by Coimbra et al. (2009) was used to sample the photoreceptor and retinal ganglion cell layer neurons.

A counting grid of a predetermined size (Table S2) was superimposed onto the retina with a randomized starting location. Each grid point that fell within the retinal outline represented a sampling location where a fraction of the area represented by that grid point was sampled using a sampling frame of predetermined size (Table S2). The number of cells marked within each sampling frame was extrapolated to estimate the number of cells located within the associated grid location (Coimbra et al., 2009). Total cell number in the retina was estimated by summing all grid locations (Eqn 1, Table 1):
(1)

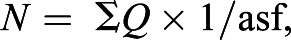
where asf is the area sampling fraction, Σ*Q* is the sum of markers counted within a frame and *N* is the total number of cells estimated within the area represented by the grid.

**Table 1.**
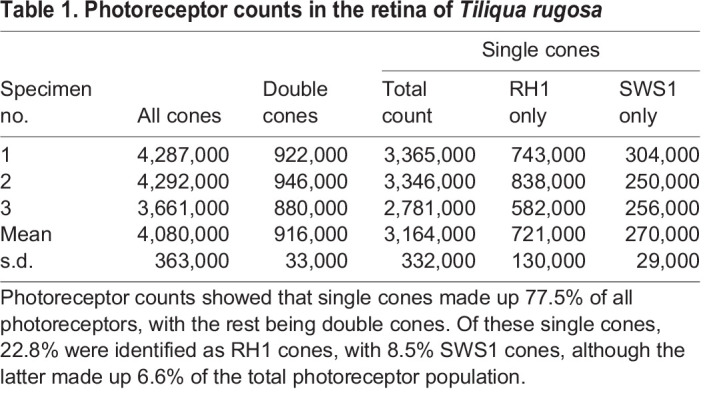
Photoreceptor counts in the retina of *Tiliqua rugosa*

A total of six retinae was used to sample retinal neurons in *T. rugosa*. Three retinae were labelled with rho-4D2 (i.e. RH1 opsin) and three retinae were labelled with sc-14363 (i.e. SWS1 opsin). Retinae labelled with rho-4D2 were also used to investigate the overall distribution and number of photoreceptors by counting and mapping all photoreceptor types, including those that were unlabelled (i.e. single and double cones).

### Generation of topographic maps

Topographic maps of photoreceptor distribution were constructed using a custom-written Matlab (MathWorks) function to determine how the distribution of photoreceptor subtypes differed across the retina. The sampling locations, retinal outline and cell locations were extracted from the xml file generated by the StereoInvestigator software (MBF Bioscience). A thin plate spline was fitted (second-order polynomial, λ=0) across sampling locations and used to interpolate the cell density at 20 µm intervals across the retina (Garza-Gisholt et al., 2014; Hemmi and Grünert, 1999). The spline reduced the effect of outlier fluctuations in the sampling, while providing a high-resolution estimate of cell density across the retina. Cell density across the retina was visualized using a combination of colour maps and contour lines, which facilitated the identification of areas of retinal specialization.

## RESULTS

### Transmittance of ocular media and absorptance of oil droplets

**Fig. 1.**
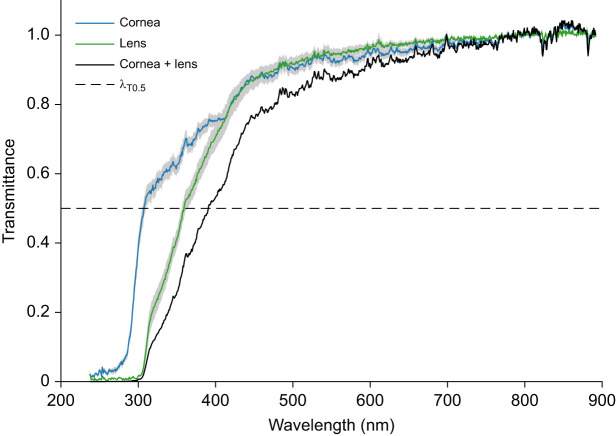
**Transmittance at the lens and cornea of *Tiliqua rugosa*.** The lens is the major optical element that limits transmission at shorter wavelengths (half-peak transmittance λ_T0.5_ at 359 nm). In contrast, the cornea transmits more light across the spectrum, with λ_T0.5_ at 307 nm. The combined optical elements achieve λ_T0.5_ at 391 nm, which would absorb more than half of all ultraviolet light entering the eye. The grey shading around the transmittance curves of the cornea and lens indicates s.e.m. Individual transmittance curves were normalized to the average transmittance value from 840 to 690 nm. *N*=6 individuals for cornea spectra, *N*=4 for lens spectra.

The lens and cornea of *T. rugosa* were clear with no noticeable colouring. The lens and the cornea reached half-peak transmittance (λ_T0.5_) at 359 and 307 nm, respectively (Fig. 1). The lens and cornea combined absorbed 50% of the light at 391 nm (Fig. 1), resulting in less than 50% of the available ultraviolet light being transmitted into the eye.

**Fig. 2.**
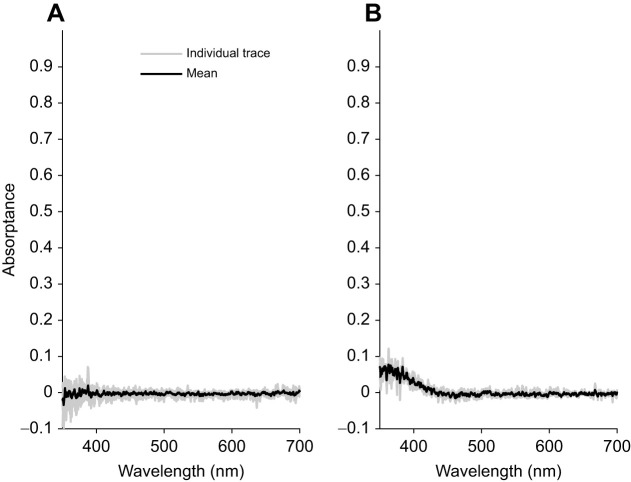
**Absorptance spectra of *T. rugosa* oil droplets.** The oil droplets were similar to the C2 (A) and C1 (B) oil droplets described in *Anolis valencienni*, where they are associated with SWS1 and SWS2 photoreceptors, respectively (Loew et al., 2002). Mean absorptance (black line) is overlayed over absorptance traces of individual measurements (grey lines); *N*=5 for A, *N*=3 for B.

Oil droplets were mainly of two types, transparent and pale yellow, with only a small number that could be reliably measured. The transparent oil droplet possessed equal absorptance throughout the spectrum, while the pale-yellow oil droplet had slightly increased absorptance below 430 nm (Fig. 2). Absorptance profiles indicate that these two types of oil droplets are analogous to C2 (Fig. 2A) and C1 (Fig. 2B) oil droplets previously described in *Anolis valencienni* (Loew et al., 2002).

### Spectral sensitivity

**Fig. 3.**
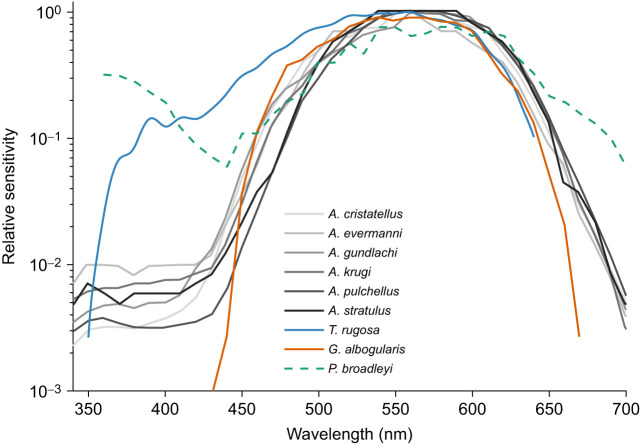
**Spectral sensitivity of the lizard eye.** The spectral sensitivity of *T. rugosa* at a stimulus frequency of 10 Hz peaks at 560 nm with a strong shoulder extending to about 390 nm (blue line). Sensitivity to short­-wavelength light is much higher than in anole lizards and diurnal geckoes, where comparable stimuli were used (10–13 Hz) (Fleishman et al., 1997; Ellingson et al., 1995). Although brief monochromatic flashes were used instead of flicker photometry to measure the spectral sensitivity of *P. broadleyi*, both it and *T. rugosa* possess much higher sensitivity to short-wavelength light than other diurnal lizards. However, unlike in *P. broadleyi*, sensitivity to ultraviolet light drops dramatically in *T. rugosa*. Note that the spectral sensitivity curve of *G. albogularis* was measured in a setup that did not transmit ultraviolet light; however, light transmission was relatively similar from ∼440 to 650+ nm, providing a reasonable comparison above 450 nm. Spectral sensitivity curves were plotted on a log scale here, unlike in other figures, to provide a clearer comparison with data gathered from Fleishman et al. (2011). Spectral sensitivity curves were normalized to the peak sensitivity.

The spectral sensitivity of *T. rugosa* recorded with a stimulus frequency of 10 Hz peaked at 562±17 nm and was dominated by long-wavelength responses (Fig. 3). The full width at half-maximum of the spectral sensitivity curve was 135 nm, ranging from 475 to 610 nm. This is much wider than the spectral sensitivity curves of eight other diurnal lizards (range 107 to 118 nm), even though they all peak in the same part of the spectrum (Fig. 3). The spectral sensitivity of *T. rugosa* was broadened relative to that of other diurnal lizards as a result of increased sensitivity at shorter wavelengths. However, unlike in *P. broadleyi*, a secondary peak in the ultraviolet region was lacking.

**Fig. 4.**
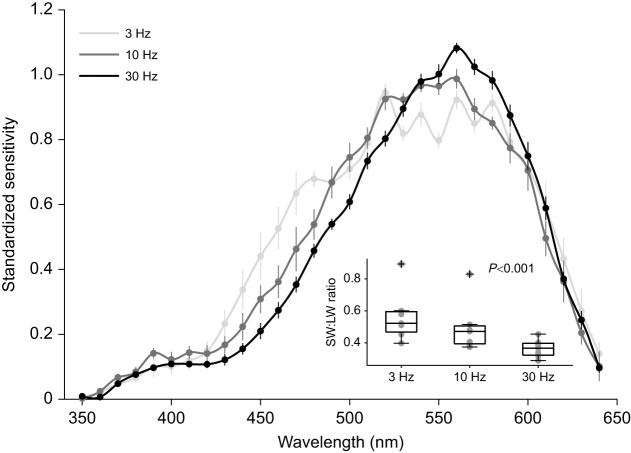
**Spectral sensitivity of the *T. rugosa* eye at different temporal frequencies.** Spectral sensitivity, measured at 3, 10 and 30 Hz, was narrower at higher temporal frequencies. Data are means±s.e.m., *N*=7 for all groups. Inset: the ratio of short-wavelength (<530 nm) to long-wavelength (>530 nm) sensitivity (SW:LW sensitivity) changed significantly across temporal frequencies (*P*<0.001), with greater short-wavelength photoreceptor contributions at lower temporal frequencies. Boxplot limits denote 25th and 75th percentiles and the area between whiskers spans ±2.7 s.d.; data points beyond this area were considered outliers and are illustrated with a plus sign.

Changing the flicker rate of the stimulus significantly changed the contributions of short-wavelength photoreceptors to the spectral sensitivity of the eye. The ratio of short- to long-wavelength sensitivity was significantly higher at 3 Hz than at 30 Hz (*P*<0.001, d.f.=19), indicating that photoreceptors sensitive to shorter wavelengths contribute significantly more to the overall response at lower temporal frequencies. Compared with the standard 10 Hz stimulus, a 3 Hz stimulus widened the spectral sensitivity curve (158 nm; Fig. 4) by 23 nm while a 30 Hz stimulus narrowed the curve (129 nm; Fig. 4) by 6 nm.

**Fig. 5.**
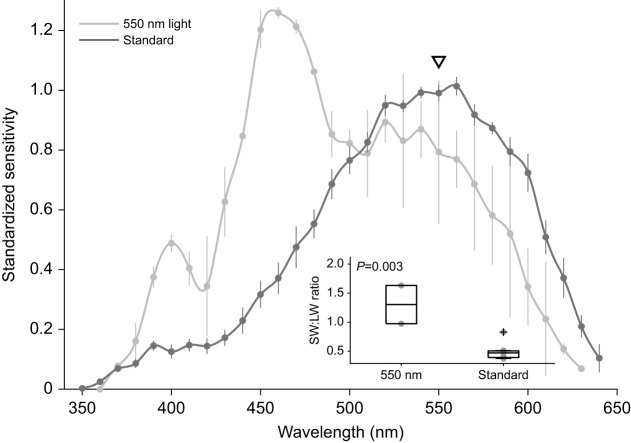
**Spectral properties of photoreceptor populations in *T. rugosa* eye.** A monochromatic light at 550 nm used to suppress LWS responses revealed contributions by a short-wavelength-sensitive cell population with a pronounced peak at 460 nm. The triangle indicates the spectral location of the 550 nm light. Data are means±s.e.m., *N*=2 individuals for the 550 nm group, *N*=7 for the standard group. Inset: the SW:LW sensitivity ratio was significantly higher with 550 nm light than with the standard 10 Hz stimulus (*P*<0.003). Boxplots as in Fig. 2.

**Fig. 6.**
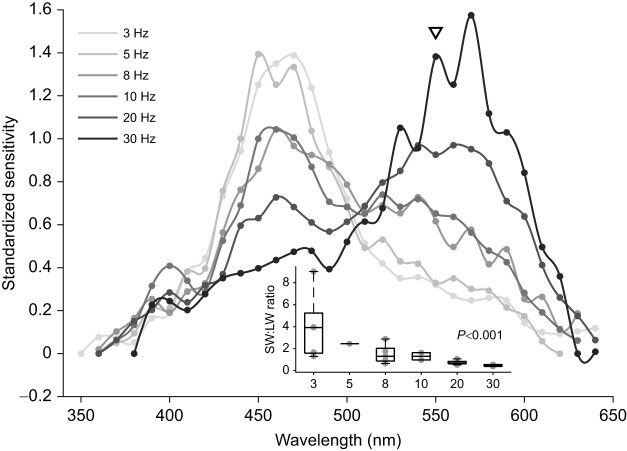
**Relative sensitivity to short-wavelength light of *T. rugosa* eye.** Sensitivity was highest at low temporal frequencies (inset; *P*<0.001), with a peak in spectral sensitivity at 470 nm revealed under 550 nm monochromatic at 3 Hz. This suggests that a blue-sensitive photoreceptor subtype drives the broadening of the spectral sensitivity of the eye at low temporal frequencies. The black triangle indicates the spectral location of the 550 nm monochromatic light. *N*=5 individuals for 3, 8 and 20 Hz, *N*=2 for 10 and 30 Hz, and *N*=1 for 5 Hz flicker rates. Boxplots as in Fig. 2.

To reveal the spectral properties of the photoreceptor populations that could be contributing to this significant change, we used monochromatic light at 550 nm. This selectively suppressed the contribution of LWS photoreceptors by reducing stimulus contrast around 550 nm and partially adapting photoreceptors that are very sensitive to the monochromatic light. At 10 Hz, the addition of the 550 nm light shifted the peak sensitivity to 460 nm (*P*=0.003, d.f.=7; Fig. 5). Under the monochromatic light, varying the temporal frequency of our stimulus significantly changed the spectral sensitivity curve (*P*<0.001, d.f.=18; Fig. 6). At slow flicker frequencies (3 Hz), the spectral sensitivity curve peaked at 470 nm and had a narrow bandwidth (72 nm). In contrast, at fast flicker frequencies (20–30 Hz), the spectral sensitivity curve was dominated by photoreceptor responses with a peak sensitivity around 550 nm (Fig. 6).

### The reflectance of blue-tongued lizards

**Fig. 7.**
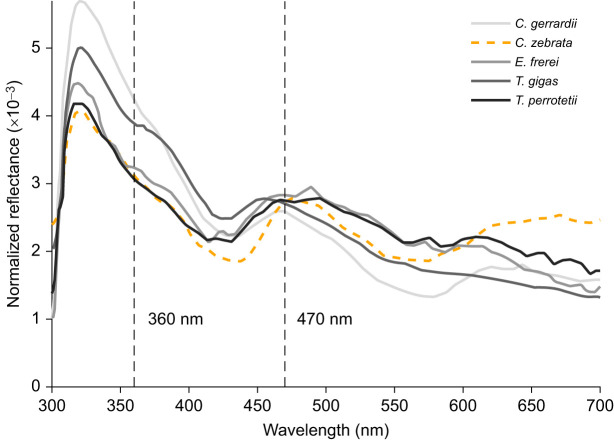
**Reflectance of blue versus pink tongues in lizards.** Normalized reflectance of pink (orange dashed line) and blue tongues (grey to black lines) of lizards (adapted from Abramjan et al., 2015). Blue tongues have a higher normalized reflectance between 360 and 470 nm than the pink tongue of *Corucia zebrata*. Reflectance curves were normalized to their respective integrals. The vertical dashed lines indicate the precise location of 360 and 470 nm on the *x*-axis.

The blue tongues of lizards reflect strongly at short wavelengths with a primary peak at approximately 320 nm and a secondary peak at approximately 460 nm (Fig. 7; Abramjan et al., 2015). The pink tongue of *C. zebrata* had a similar bimodal reflectance profile but the balance of reflectance between short and long wavelength was shifted towards longer wavelength in *C. zebrata* (Fig. 7).

### Opsin sequences

**Fig. 8.**
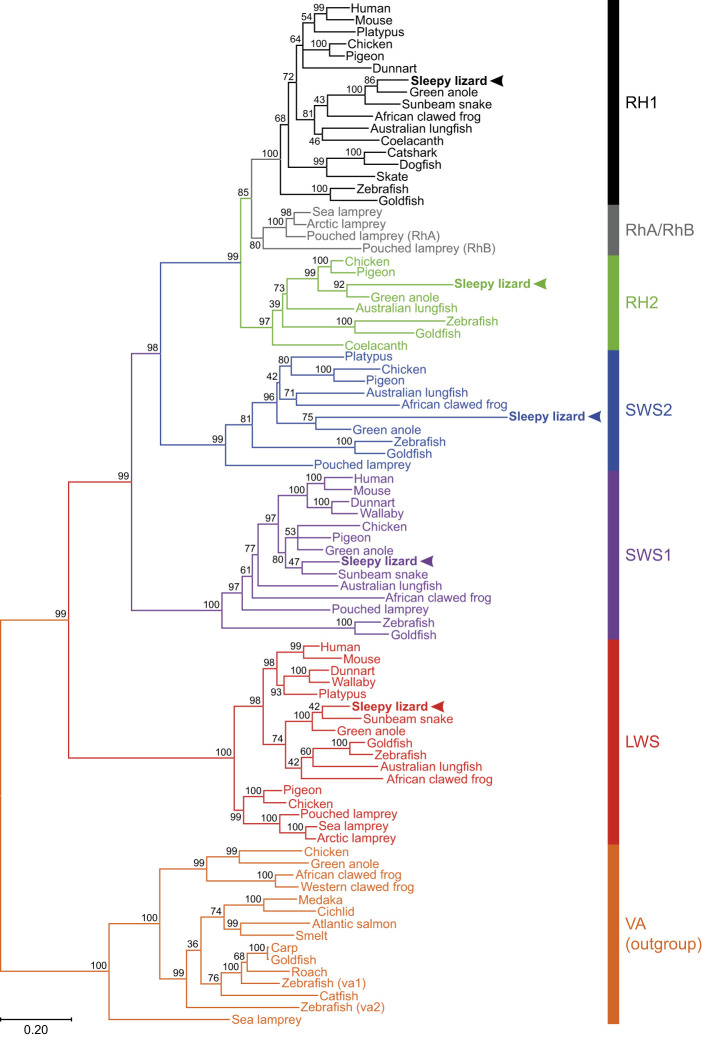
**Opsin genes in vertebrates.** The evolutionary history was inferred by using the Maximum Likelihood method and General Time Reversible model (Nei and Kumar, 2000). The tree with the highest log likelihood (−38890.45) is shown. The percentage of trees for which the associated taxa clustered together is shown next to the branches. Initial trees for the heuristic search were obtained automatically by applying Neighbour-Join and BioNJ algorithms (Saitou and Nei, 1987) to a matrix of pairwise distances estimated using the Maximum Composite Likelihood (MCL) approach (Tamura and Nei, 1993), and then selecting the topology with superior log likelihood value. A discrete gamma distribution was used to model evolutionary rate differences among sites (5 categories; +*G*, parameter=0.9476). The rate variation model allowed for some sites to be evolutionarily invariable ([+*I*], 10.93% sites). The tree is drawn to scale, with branch lengths measured in the number of nucleotide substitutions per site (indicated by the scale bar). This analysis involved 85 nucleotide sequences, where the five *T. rugosa* visual photopigment genes (*LWS*, *SWS1*, *SWS2*, *RH2* and *RH1*; arrowheads) have the following GenBank accession numbers: ON637851–ON637855. Vertebrate ancient (VA) opsin sequences were collectively used as an outgroup. Codon positions included were 1st+2nd+3rd+Noncoding. All positions with <95% site coverage were eliminated, i.e. <5% alignment gaps, missing data and ambiguous bases were allowed at any position (partial deletion option). There were a total of 903 positions in the final dataset. Evolutionary analyses were conducted in MEGA11 (Tamura et al., 2021).

Five visual opsin genes were found to be expressed in the retina of the sleepy lizard (Genbank accession numbers ON637851–ON637855). Sequence alignment and phylogenetic analyses confirmed them to be true orthologues of *LWS*, *SWS1*, *SWS2*, *RH2* and *RH1* opsin genes identified in other vertebrates (Fig. 8; Table S2). Upon closer inspection, it was possible to examine 30 out of the 34 known tuning sites across the five opsin genes and of these, only one tuning site (in the SWS2 opsin) differed from those in the green anole (*Anolis carolinensis*). There was a high sequence similarity between the opsins of the sleepy lizard and the green anole (Fig. S2).

### Retinal topography

Five photoreceptor counts were made and mapped separately, including total photoreceptors, single cones, double cones, RH1 cones and SWS1 cones (the last two were isolated immunohistochemically; Fig. S1A,B). The total photoreceptor population comprised 77.5% single cones and 22.5% double cones (Table 1). No labelling with antibodies specific to RH1 or SWS1 was observed in double cones, suggesting that principal and accessory members of the double cones contain SWS2, RH2 or LWS opsins. RH1 photoreceptors comprised 22.8% of single cones and 17.7% of all photoreceptors. In contrast, the number of SWS1 cones was significantly lower at 8.5% of single cones and 6.6% of the total photoreceptor population (Table 1).

**Fig. 9.**
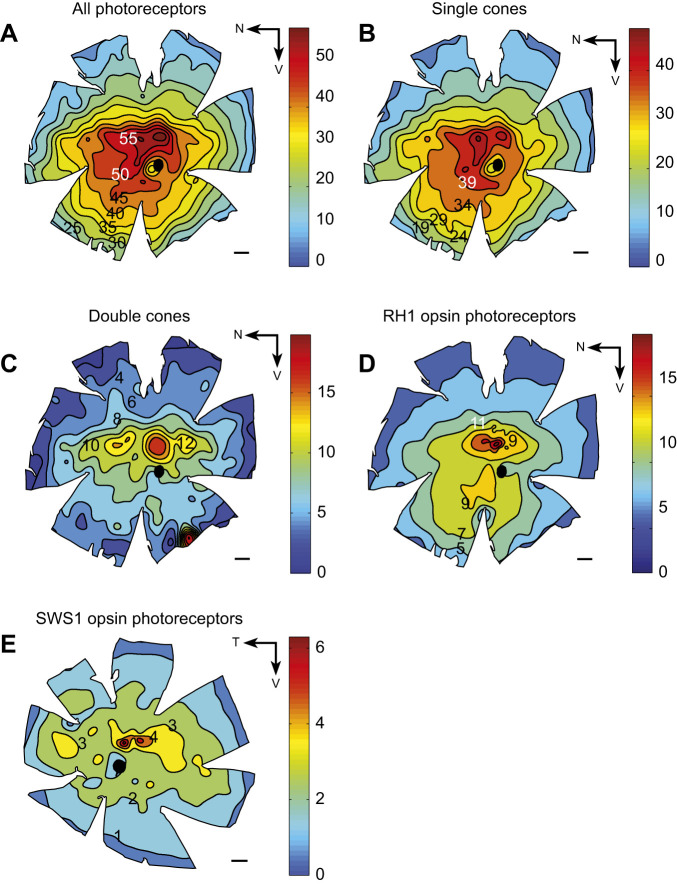
***Tiliqua rugosa* photoreceptor population.** All photoreceptor populations showed peak density in the retinal centre, with an area centralis and ventral wedge of high photoreceptor density (A), single cones (B), double cones (C) and RH1-expressing cones (D). SWS1-labelled photoreceptors (E) were fewer in number and showed a shallower slope of density increase towards the centre. N, nasal retina; T, temporal retina; V, ventral retina. Scale bars: 1 mm across all maps. Black circles in the middle of the maps indicate the optic nerve head. Iso-density lines and coloured bars indicate density in cells mm^−2^ (×1000).

All single cones and double cones had similar distributions across the retina. The density increased steadily towards the retinal centre, forming an area centralis. There was a slight asymmetry with a higher number of photoreceptors in the ventral compared with the dorsal retina (Fig. 9A–D). Although no clear horizontal visual streak was observed, relatively high densities (between 35,000 to 55,000 photoreceptors mm^−2^) were maintained across the naso-temporal axis of the retina at the retinal meridian (Fig. 9A–D). SWS1 single cones also adopted a concentric increase in density towards the central retina but had a much shallower gradient, with densities ranging from 2000 to 5000 SWS1 photoreceptors mm^−2^ in the retinal centre (Fig. 9C).

## DISCUSSION

Our findings reveal that *T. rugosa* has an elevated sensitivity at shorter wavelengths, which is distinct from other diurnal lizards. Here, we discuss potential drivers for these differences such as spectral tuning and photoreceptor abundance, their ecological benefits and future research directions.

### Sensitivity to ultraviolet light differs from that of other diurnal lizards

A close look at the ultraviolet portion of the spectral sensitivity curves of diurnal lizards studied to date (Fig. 3) reveals that a secondary peak occurs between 350 and 360 nm followed by a slight reduction in sensitivity at 370 to 380 nm. This is typically associated with the peak sensitivity of SWS1 photoreceptors which, in diurnal lizards, typically ranges from 358 to 367 nm. Influences from the beta peak of LWS and MWS photoreceptors are likely to be minimal or non-existent in the short-wavelength region because of their association with oil droplets, which typically absorb most/all light below ∼470 nm as described in the anole lizard retina (Loew et al., 2002). In contrast to most diurnal lizards, the cordylid lizard, *Platysaurus broadleyi*, shows elevated sensitivity to ultraviolet at 360 nm, which has been associated with an increased abundance of UVS photoreceptors (Fig. 3, green dashed line; Fleishman et al., 2011). However, in *T. rugosa*, there appears to be no ultraviolet peak, but a sharp drop after 380–390 nm. There is no conspicuous peak in sensitivity to ultraviolet light even when using stimuli that should simultaneously suppress LWS contributions while increasing the contributions of SWS photoreceptors (550 nm monochromatic light+low temporal frequencies; Fig. 6). This suggests that despite the presence of SWS1, which is typically associated with UVS photoreceptors in diurnal lizards, UVS photoreceptors may be absent in *T. rugosa*. This could be caused by a shift of the spectral sensitivity of SWS1 photoreceptors towards longer wavelengths.

### Red-shifting the spectral sensitivity of the SWS1 photoreceptor

#### Single-site amino acid substitution?

Large shifts in the spectral sensitivity of SWS1 photoreceptors from ultraviolet to violet are commonly associated with single-site amino acid substitutions at position 86 of the SWS1 photopigment (Hauser et al., 2014). In freshwater (*Helicops modestus*) and sea (*Aipysurus* spp*.*, *Epiocephalus* spp*.*, *Hydrophis* spp*.*) snakes, the amino acid phenylalanine is replaced by valine, serine, cysteine or tyrosine at position 86 of the SWS1 photopigment, thereby shifting its spectral sensitivity towards longer wavelengths (Hauzman et al., 2017; Simões et al., 2020). All diurnal lizards studied to date possess phenylalanine at site 86, which results in ultraviolet-sensitive photopigments; however, in contrast to aquatic snakes, no amino acid substitutions have yet been reported. While we have been unable to confirm the amino acid identity at site 86 of the SWS1 photopigment in *T. rugosa*, the conspicuous lack of ultraviolet sensitivity in the ERGs suggest that phenylalanine has been replaced by another amino acid to produce violet-sensitive (VS) photoreceptors instead of UVS photoreceptors.

#### Alternative spectral tuning mechanisms and why they are less likely

Mechanisms such as changes from an A_1_ photopigment to an A_2_ photopigment, and the filtering effects of ocular media and oil droplets, may also shift the spectral sensitivity of retinal photoreceptors. However, we describe below how their potential to shift the spectral sensitivity of a hypothetical UVS photoreceptor is limited and would not be congruent with the lack of a peak in sensitivity in the ultraviolet region of Fig. 6 for low temporal frequency stimuli.

Transitions from A_1_ to A_2_ chromophores are commonly used in fish to shift the spectral sensitivity of their photopigments to longer wavelengths (Bowmaker, 1995; Whitmore and Bowmaker, 1989); however, in diurnal lizards, this is relatively rare, with the adoption of pure A_2_ photopigments only observed in the green anole, *A. carolinensis* (Kawamura and Yokoyama, 1998)*.* SWS, MWS and LWS photopigment sensitivity of the green anole was shifted towards longer wavelengths, but the UVS photoreceptor spectral sensitivity remained unchanged by the adoption of the A_2_ chromophore (Loew et al., 2002). In the unlikely event that *T. rugosa* uses pure A_2_ chromophores, we therefore expect little to no effect on the spectral sensitivity of potential UVS photoreceptors given the high sequence similarity of photopigments between green anoles and *T. rugosa*.

Oil droplets associated with SWS1 photoreceptors tend to be transparent in all measurements in lizards to date, and we have identified a transparent oil droplet likely to be similarly associated in *T. rugosa*. We therefore expect little to no effect of oil droplets on the SWS1 photoreceptors. In contrast to the oil droplets, the λ_T0.5_ of the lens is long wave-shifted in *T. rugosa* (356 nm) compared with diurnal lizards (∼320 nm; Pérez i de Lanuza and Font, 2014) and would absorb more than half of the ultraviolet light entering the eye. On their own, the cornea and lens of *T. rugosa* combined would shift the λ_max_ of a typical diurnal lizard SWS1 photoreceptor from ∼365 to 377 nm; however, this would have the effect of substantially reducing the photon capture ability of such a SWS1 cone (see Fig. 6).

### Short-wavelength sensitivity is greatly enhanced by multiple factors

#### The role of SWS2 photoreceptors

Despite several lines of evidence suggesting that *T. rugosa* do not possess a UVS photoreceptor, they are up to an order of magnitude more sensitive than other diurnal lizards between 360 and 530 nm (Fig. 3) as a result of the broadness of their spectral sensitivity curve. The effect of adjusting the temporal frequency of our stimulus on the width of the spectral sensitivity curve suggests that the wider spectral sensitivity curve of *T. rugosa* is partially mediated by a population of photoreceptors that favour low temporal frequencies (Fig. 4). Peak sensitivities between 450 and 470 nm under 550 nm monochromatic light at low temporal frequencies suggest that these are SWS2 photoreceptors. This is supported by the λ_max_ of SWS2 photoreceptors at similar wavelengths in other lizards (Osorio, 2019) and the well-established high sensitivity of this photoreceptor type to low-frequency stimuli across taxa including tiger salamanders, goldfish, ants and primates (Howlett et al., 2017; Ogawa et al., 2015; Rieke and Baylor, 2000; Skorupski and Chittka, 2010; Tailby et al., 2008). An increased abundance of SWS2 photoreceptors in *T. rugosa* could explain the enhanced short-wavelength sensitivity observed here. This is different to the suspected increased abundance of SWS1 photoreceptors in *P. broadleyi*, which is thought to enhance sensitivity to ultraviolet light but leaves a dip in sensitivity to blue light (∼450 nm) (Fleishman et al., 2011; Martin et al., 2015). However, it is clear that the SWS2 photoreceptors alone cannot explain the relatively broad spectral sensitivity curve of *T. rugosa* as the curve remains broader than that of other diurnal lizards even under conditions (30 Hz; Figs 4 and 6) where the response of SWS2 photoreceptors was greatly attenuated. It is worth noting that a red-shifted SWS1 opsin would have a greater overlap with the neighbouring SWS2 opsin and would therefore increase the quantal catch of the eye where the spectra of the photoreceptors overlap.

#### Oil droplets associated with MWS and LWS photoreceptors

Typically, MWS and LWS photoreceptors are associated with green (G), orange (O) or yellow (Y) oil droplets, which absorb most of the light below 470 nm, thereby reducing their contribution to short-wavelength sensitivity (Barbour et al., 2002; Crescitelli, 1972; Loew et al., 2002; Martin et al., 2015). However, our findings and the previous report by New et al. (2012) suggest that *T. rugosa* only has two types of oil droplets, both of which transmit most of the light below 470 nm. New et al. (2012) further reported that pale-yellow oil droplets are associated with single cones and the principal member of the double cone, which are typically LWS photoreceptors (Crescitelli, 1972). This suggests that in *T. rugosa*, at least the LWS photoreceptor, if not both the MWS and the LWS photoreceptors, is associated with pale-yellow oil droplets. This would result in significantly greater sensitivity to shorter wavelength light and could partially explain the broad spectral sensitivity curve of *T. rugosa* relative to that of other diurnal lizards.

### Insights from the abundance and distribution of photoreceptors

#### Photoreceptor subtype abundance

The typical abundance of SWS1 photoreceptors in birds and mammals is around 5–10% (Hart, 2001; Hunt and Peichl, 2014; Kram et al., 2010). Similar estimates have been observed in turtles (6%, *Pseudemys scripta*) and anole lizards (5%, *A. sagrei*) where the SWS1 opsin is maximally sensitive to ultraviolet (Fleishman et al., 2011; Grötzner et al., 2020). In *Platysaurus broadleyi* which possesses enhanced ultraviolet sensitivity, ERG data and transparent oil droplet counts suggests that 16.5% of photoreceptors are UVS photoreceptors, which are likely to contain the SWS1 opsin. In contrast, only 6.6% of photoreceptors in the retina of *T. rugosa* express SWS1, indicating that an increased abundance of SWS1 is not driving enhanced sensitivity to short wavelengths in this species. Instead, the abundance of SWS1 photoreceptors is similar to the broadly conserved percentages of SWS1 photoreceptors found across taxa.

SWS2 photoreceptors, identified through oil droplet counts in birds and turtles, comprise 10–15% of the total population of photoreceptors, which is 1–1.5 times the number of SWS1 photoreceptors (Grötzner et al., 2020; Hart, 2001). Assuming that those proportions are preserved in *T. rugosa*, we can assume that at least 6.6–9.9% of photoreceptors will express SWS2 opsins or a higher percentage if the abundance of SWS2 photoreceptors is elevated in this species.

The presence of RH1 in cone-like cells has been widely reported in other diurnal lizards and snakes, but this is the first time the proportion of RH1 cones has been accurately quantified over the entire retina of a squamate. Our finding that 17.7% of photoreceptors express the RH1 opsin suggests that these transmuted cells (Walls, 1942) make a significant contribution to the response of the whole eye. While we successfully labelled and quantified the abundance of RH1 photoreceptors, it remains unknown what percentage of the photoreceptor population is composed of RH2 photoreceptors.

Although LWS photoreceptors were not labelled in this study, previous reports by New et al. (2012) indicate that the LWS opsin is not co-expressed with the SWS1 or RH1 opsins in the retina of *T. rugosa*. The expression of the LWS opsin in the accessory and principal members of the double cones, as well as in a subset of single cones, is well established in diurnal lizards (Loew et al., 2002). This expression pattern is also present in birds and turtles, suggesting a well-preserved feature that is probably repeated in the retina of *T. rugosa*. Given that double cones comprise 22% of the total photoreceptor population, it is highly likely that LWS photoreceptors are the most abundant photoreceptor subtype in the retina of *T. rugosa*. This would align well with the spectral sensitivity of the whole eye peaking at ∼560 nm observed in this study and in other studies of diurnal lizards (Ellingson et al., 1995; Fleishman et al., 1997).

#### Photoreceptor subtype topographic distribution

The high density of single cones and double cones which persists towards the ventral periphery of the retina suggests that the dorsal visual field is spatially sampled at a higher resolution than the ventral visual field of *T. rugosa*. This is likely, as the head of *T. rugosa* is very close to the ground with most ecologically relevant interactions likely to occur at eye level or within the dorsal visual field. Similar patterns which mirror our observations are found in the retina of artiodactyls, where height is coupled to the density of photoreceptors in the dorsal retina, with taller artiodactyls possessing higher densities of photoreceptors in their dorsal retina (Schiviz et al., 2008). However, unlike other types of photoreceptors mapped here, the SWS1 photoreceptors are rather uniformly distributed. Because of the low abundance of SWS1 photoreceptors, they may be less relevant to high acuity vision and, therefore, they may not be under the same selective pressure which produces dorso-ventral asymmetries in the distribution of other photoreceptor types.

Up to now, the distribution of RH1 photoreceptors in squamates has remained unmapped. The dorsoventral asymmetry in the distribution of RH1 photoreceptors is not dissimilar to previous findings in passerine birds and various mammals (Coimbra et al., 2015; Kryger et al., 1998; Müller and Peichl, 1989). However, unlike in the passerines and raptors (Coimbra et al., 2015; Mitkus et al., 2017), the peak density of RH1 photoreceptors coincides with the peak of all photoreceptors and there are no retinal areas where the RH1 opsin is not expressed. In *A. carolinensis*, RH1 photoreceptors are expressed even in the fovea, whereas in bird foveae, RH1 photoreceptors are completely absent (Coimbra et al., 2015; McDevitt et al., 1993; Mitkus et al., 2017). While *T. rugosa* does not possess a fovea, the difference in the retinal distribution of these cells between birds and squamates suggests that RH1 photoreceptors play a different role in their respective visual systems (Schott et al., 2016; Zhang et al., 2006).

### Ecological relevance of higher sensitivity to short wavelengths

*Tiliqua rugosa* belongs to a group of lizards that possess blue tongues, which they are suspected of using to deter predators and to avoid aggressive male-to-male interactions (Abramjan et al., 2015; Badiane et al., 2018). Despite the similar reflectance profile of blue and pink tongues, blue tongues tend to reflect relatively more short-wavelength light (Fig. 7) as the pigment in their tongues probably absorbs longer wavelength light (Abramjan et al., 2015). Previous visual models report that blue tongues are more conspicuous to species of the *Tiliqua* genus than to their potential aerial predators (Abramjan et al., 2015); however, this assumes that *Tiliqua* spp*.* possess similar enhanced ultraviolet sensitivity to *P. broadleyi*. Instead, our findings show that *T. rugosa* possesses enhanced sensitivity over a broader region of the spectrum and may be missing UVS photoreceptors. While this may still make the blue tongue, which is common in this genus, more conspicuous to its conspecifics than to its predators, it suggests that signal detection is fundamentally different to what has been previously reported.

### Conclusions

The eye of *T. rugosa* is up to an order of magnitude more sensitive to short wavelengths than are those of other diurnal lizards, suggesting that the detection of short-wavelength light plays a relatively important role in the ecology of this species. This enhanced sensitivity appears to be achieved by multiple factors which involve blue photoreceptors tuned to slow temporal frequencies, the absence of yellow and green oil droplets and potentially red-shifted SWS1 photoreceptors. While our findings demonstrate the effect of these factors on the overall sensitivity of the eye, the precise way in which these mechanisms are being implemented in the retina of *T. rugosa* remains unclear. Additional studies should fully sequence the tuning sites of the SWS1 opsin, rigorously characterize the association between oil droplets and photoreceptor types and accurately estimate the abundance of SWS2 photoreceptors. While this enhanced sensitivity to short wavelengths may facilitate the detection of the blue tongue common in the *Tiliqua* genus, it is unclear whether the eye adapted to an existing signal to facilitate detection or whether tongue colour adapted to pre-existing high short-wavelength sensitivity by becoming blue. The great difference between the spectral sensitivity of *T. rugosa* and that of other diurnal lizards suggests that the visual system of this species and possibly that of its close relatives may be under fundamentally different phylogenetic and ecological pressure. This understudied clade of diurnal lizard therefore offers us a unique opportunity to dissect the phylogenetic and environmental pressures that direct the co-evolution of spectral sensitivity and ecologically relevant visual cues.

## Supplementary Material

Supplementary information
