# Supplementary material for: Enhanced short-wavelength sensitivity in the blue-tongued skink Tiliqua rugosa
Source: J Exp Biol. 2022 Jun 13;225(11):jeb244317. doi: 10.1242/jeb.244317 (PMC9234500; doi:10.1242/jeb.244317)
Supplement: Supplementary information [file jexbio-225-244317-s1.pdf]

Supplementary Materials and methods

Immunohistochemistry and antibody specificity

The use of immunohistochemistry to label photoreceptor subtypes in the retina of T. rugosa was limited to antibodies where high opsin specificity had been previously demonstrated. As such, only SWS1 and RH1 opsins could be successfully immunohistochemically labeled.

The sc-14363 goat polyclonal antibody (Santa Cruz Biotechnology, USA) was raised against the epitope of the human S cone opsin and has been successfully used to label SWS1 opsins in birds (Nießner et al., 2011). This is mainly due to the high similarity of amino acid sequences between the S opsin epitope of mammals and the SWS1 opsin epitope of birds (Hart and Hunt, 2007) and low epitope sequence identity to other opsins (Nießner et al., 2011). Hence, labeling of SWS1 cones by sc-14363 is highly specific and, in birds at least, shows no overlap with labeling by JH492 antibody that detects LWS-derived photopigments (Nießner et al., 2011).

The rho-4D2 mouse monoclonal antibody was raised against the N-terminus of bovine rhodopsin and has been successfully used to specifically label RH1 opsins in several lizard species, including the American chameleon (Anolis carolinensis), the chameleon Chameleo chameleon and the sleepy lizard, Tiliqua rugosa (McDevitt et al., 1993, New et al., 2012).

Retinae were incubated overnight without agitation to minimize tissue damage in a solution of 0.3% Triton X100 in 0.1 M PB (pH 7.2-7.4), 5% normal rabbit serum (rho-4D2) or 5% normal donkey serum (sc-14363) and the primary antibody (1:500 for both antibodies). Prior to being incubated with a biotinylated secondary antibody for 2 h, retinae were rinsed in 0.1 M PB buffer three times for 5 min each, before being transferred to a solution containing an avidin-biotin complex (Vectastain, ABC kit, Vector Laboratories, USA), incubated for 1 h, then rinsed three times (5 min each) in 0.1 M PB. Opsin immunoreactivity was visualized via a horseradish peroxidase (HRP) and H2O2 reaction as recommended in the peroxidase substrate kit (SK4700, Vector Laboratories, USA). After sufficient signal development, the reaction was stopped by rinsing the retinae three times (5 min each) in 0.1M PB, before being mounted in 80% glycerol in 0.1M PB plus 0.1% sodium azide.

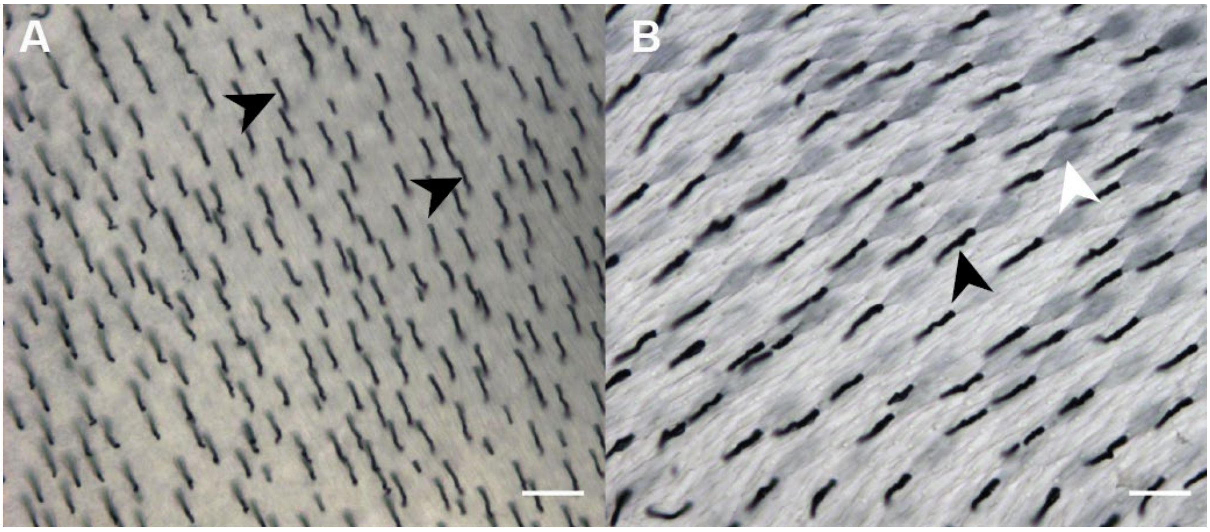

Fig. S1. Rho-4D2 and sc-14363 stained outer segments of RH1 (A) and SWS1 (B) in the retina of *Tiliqua rugosa*. Black arrowheads indicate the outer segments, and the white arrowhead indicates the inner segment of photoreceptors. Scale bars = 20 µm.

Journal of Experimental Biology • Supplementary information

Fig. S2. Codon-matched alignment showing partial amino acid sequences for all five visual opsins expressed in the retina of the sleepy lizard, T. rugosa compared to orthologues identified in the green anole, Anolis carolinensis.

```
                            10        20        30        40        50        60
Green anole RH1     -------------------MNGTEGQNFYVPMSNKTGVVRNPFEYPQYYLADPWQFSALA
Sleepy lizard RH1   -----------------------------------TGVVRSPFEYPQYYLAEPWQYSALA
Green anole RH2     -------------------MNGTEGINFYVPLSNKTGLVRSPFEYPQYYLAEPWKYKVVC
Sleepy lizard RH2   ------------------------------------------------------------
Green anole SWS2    ----------MQKSRPDSRDNLPEDFFIPVPLDVANITTLSPFLVPQTHLGNPSLFMGMA
Sleepy lizard SWS2  ---------------------------------------------PQTHLGSPGLFMGMA
Green anole SWS1    ------------------------MSGQEDFYLFENISSVGPWDGPQYHIAPMWAFYFQT
Sleepy lizard SWS1  ------------------------------------------------------------
Green anole LWS     MAGTVTEAWDVAVFAARRRNDEDDTTRDSLFTYTNSNNTRGPFEGPNYHIAPRWVYNITS
Sleepy lizard LWS   --------------------------------------TRGPFEGPNFHIAPRWVYHLTS

                            70        80        90       100       110       120
Green anole RH1     AYMFLLILLGFPINFLTLFVTIQHKKLRTPLNYILLNLAVANLFMVLMGFTTTMYTSMNG
Sleepy lizard RH1   AYMFLLILLGLPINFLTLFVTIQHKK----------------------------------
Green anole RH2     CYIFFLIFTGLPINILTLLVTFKHKKLRQPLNYILVNLAVADLFMACFGFTVTFYTAWNG
Sleepy lizard RH2   ----------------------KHKKLRQPLNYILVNLAVADLFMACFGFTVTFYTAWNG
Green anole SWS2    AFMFILIVLGVPINVLTIFCTFKYKKLRSHLNYILVNLSVSNLLVVCVGSTTAFYSFSNM
Sleepy lizard SWS2  AFMFMLVVLGVPINALTIFCTFKYKKLRSHLNYILVNLAVSNLLVVCIGSVTAFYSFSQM
Green anole SWS1    AFMGFVFFAGTPLNAIILIVTVKYKKLRQPLNYILVNISFAGFLFCTFSVFTVFMASSQG
Sleepy lizard SWS1  ------------------------------------------------------------
Green anole LWS     VWMIFVVIASIFTNGLVLVATAKFKKLRHPLNWILVNLAIADLGETVIASTISVINQISG
Sleepy lizard LWS   LWMIFVVVASVFTNGLVLVATAKFKKLRHPLNWILVNLAIADLGETVIASTISVINQLFG

                           130       140       150       160       170       180
Green anole RH1     YFIFGTVGCNIEGFFATLGGEMGLWSLVVLAVERYVVICKPMSNFRFGETHALIGVSCTW
Sleepy lizard RH1   ----------------TMGGEIALWSLVVLAVERYVVVCKPMSNFRFSETHAIMGVSFTW
Green anole RH2     YFIFGPIGCAIEGFFATLGGQVALWSLVVLAIERYIVVCKPMGNFRFSATHALMGISFTW
Sleepy lizard RH2   YFIFGPIGCAVEGFFATLGGQVALWSLVVLAIERYIVICKPMGNFRFSATHALMGIAFTW
Green anole SWS2    YFSLGPTACKIEGFSATLGGMVSLWSLAVVAFERYLVICKPLGNFTFRGTHAIIGCAVTW
Sleepy lizard SWS2  YFALGPTACKIEGFSATLGGMVSLWSLAVVAFERFLVICKPLGNFTFRGTHAIVGCIITW
Green anole SWS1    YFFFGRHVCAMEAFLGSVAGLVTGWSLAFLAFERYIVICKPFGNFRFNSKHALLVVAATW
Sleepy lizard SWS1  ----------------------------------------PFGNFRFNSKHALLVVAATW
Green anole LWS     YFILGHPMCVLEGYTVSTCGISALWSLAVISWERWVVVCKPFGNVKFDAKLAVAGIVFSW
Sleepy lizard LWS   YFILGHPMCVLEGYTVSACGITALWSLAIISWERWVVVCKPFGNIKFDAKLAMLGIVFSW

                           190       200       210       220       230       240
Green anole RH1     IMALACAGPPLLGWSRYIPEGMQCSCGVDYYTPTPEVHNESFVIYMFLVHFVTPLTIIFF
Sleepy lizard RH1   MMALACAGPPLLGWSRYIPEGMQCSCGVDYYTPNPEVHNKSFVIYMFVVHFVTPLSII--
Green anole RH2     FMSFSCAAPPLLGWSRYIPEGMQCSCGPDYYTLNPDYHNESYVLYMFGVHFVIPVVVIFF
Sleepy lizard RH2   FMALACACPPLFGWSRYIPEGMQCSCGPDYYTHNPDYHNESYVLYMFVIHFVIPVVVIFF
Green anole SWS2    MFGLAASLPPLFGWSRYIPEGLQCSCGPDWYTTENKWNNESYVIFLFCFCFGVPLSVIIF
Sleepy lizard SWS2  IIGLVASLPPLFGWSRYIPEGLQCSCGPDWYTTNNKWNNESYVMFLFCFCFGVPLSVIVF
Green anole SWS1    FIGIGVSIPPFFGWSRYIPEGLQCSCGPDWYTVGTKYKSEYYTWFLFIFCFIVPLTLIIF
Sleepy lizard SWS1  FIGIGVSIPPFFGWSRFIPEGLQCSCGPDWYTVGTKYKSEYYTWFLFIFCFIVPLTLIIF
Green anole LWS     VWSAVWTAPPVFGWSRYWPHGLKTSCGPDVFSGSDDPGVLSYMIVLMITCCFIPLAVILL
Sleepy lizard LWS   VWSCAWTAPPIFGWSRYWPHGLKTSCGPDVFSGSSDPGVQSYMVVLMITCCFFPLSIIIL

                           250       260       270       280       290       300
Green anole RH1     CYGRLVCTVKAAAAQQQESATTQKAEREVTRMVVIMVISFLVCWVPYASVAFYIFTHQGS
Sleepy lizard RH1   ------------------------------------------------------------
Green anole RH2     SYGRLICKVREAAAQQQESASTQKAEREVTRMVILMVLGFLLAWTPYAMVAFWIFTNKGV
Sleepy lizard RH2   SYGRLICKVREAAAQQQESASTQKAEKEVTRMVILMVLGFLLAWTPYAVVAFWIFTNKGA
Green anole SWS2    SYGRLLLTLRAVAKQQEQSATTQKAEREVTKMVVVMVMGFLVCWLPYASFALWVVTHRGE
Sleepy lizard SWS2  SYGRLLLTLRAVAKQQEQSATTQKAEREVTKMVIVMVMGFLVCWLPYASFSLWIVTHRGE
Green anole SWS1    SYSQLLGALRAVAAQQQESATTQKAEREVSRMVVVMVGSFCLCYVPYASLAMYMVNNRDH
Sleepy lizard SWS1  SYSQLLGALRAVAAQQQESATTQKAEREVSRMVVVMVGSFCLCYVPYAALAMYMVNNRNH
Green anole LWS     CYLQVWLAIRAVAAQQKESESTQKAEKEVSRMVVVMIIAYCFCWGPYTVFACFAAANPGY
Sleepy lizard LWS   CYLQVWMAIRAVAAQQKESESTQKAEREVSRMVVVMILAYIFCWGPYTIFACFAAANPGY
```

Journal of Experimental Biology • Supplementary information

```
                              310       320       330       340       350       360
Green anole RH1     DFGPVFMTIPAFFAKSSAIYNPVIYILMNKQFRNCMIMTLCCGKNPLG-DEDTSAG---T
Sleepy lizard RH1   ------------------------------------------------------------
Green anole RH2     DFSATLMSVPAFFSKSSSLYNPIIYVLMNKQFRNCMITTICCGKNPFG-DEDVSSSVSQS
Sleepy lizard RH2   EFSATFMSVPAFFSKSSSLYNPVIYVLMNKQFRNCMITTICCGKNPFG-DDDVSSTVSQS
Green anole SWS2    PFDVRLASIPSVFSKASTVYNPVIYVLMNKQFRSCMLKLIFCGKSPFGDEDDVSGS-SQA
Sleepy lizard SWS2  PFDVSLASIPSVFSKASTVYNPIIYVFMNKQFRSCMMKLVFCGKSPFGDEDDVSGS-SQA
Green anole SWS1    GLDLRLVTIPAFFSKSSCVYNPIIYCFMNKQFRACIL-ETVCGKPMS-DESDVSSS-AQ-
Sleepy lizard SWS1  GIDLRLVTIPAFFSKSACVYNPIIYCFMNKQFRGCIM-ETVCGKPMT-DESDVSSS-AQ-
Green anole LWS     AFHPLAAALPAYFAKSATIYNPIIYVFMNRQFRNCIM--QLFGKKVD-DGSELSST--S-
Sleepy lizard LWS   AFHPLAAALPAFFAKSATIYNPIIYVFMNRQFRNCI------------------------


                              370
Green anole RH1     KTETSTVSTSQVSPA*
Sleepy lizard RH1   ----------------
Green anole RH2     KTEVSSVSSSQVSPA*
Sleepy lizard RH2   KT--------------
Green anole SWS2    -TQVSSVSSSQVSPA*
Sleepy lizard SWS2  -TQVSSVS--------
Green anole SWS1    KTEVSSVSSSQVSPS*
Sleepy lizard SWS1  KTEVSS----------
Green anole LWS     RTEVSSVSNSSVSPA*
Sleepy lizard LWS   ----------------
```

Journal of Experimental Biology • Supplementary information

Table S1. Primers used to isolate and amplify sleepy lizard opsin gene sequences from retinal cDNA (Davies et al., 2009, Knott et al., 2013, Hart et al., 2016).

Name	Sequence (5' to 3')
AOASF1	CGCGAGAGATACATNGTNRTNTGYAARCC
AOASF2	ATTTTAGAAGGTCTGCCRGWSNTCNTGYGG
AOASR1	ATTGGTCACCTCCTTYTCNGCYYTYTGNGT
AOASR2	CCCGGAAGACGTAGATGANNGGRTTRWANA
DIAPLMF1	AAGCGTATTYAYTTAYACCRACASCAACAA
DIAPLMF2	AGTGTCATCAACCAGWTCTYBGGSTAYTTC
DIAPLMR1	CATCCTBGACACYTCCYTCTCVGCCTTCTG
DIAPLMR2	CATCATCCACTTTYTTSCCRAASAGCTGCA
DIAPS1F1	TCCCATGTCCGGAGAVGAVGABTTYTACCT
DIAPS1F2	GGCCTTCGARCGHTACATYGTYATCTGCAA
DIAPS1R1	CACCACSACCATSCGVGASACCTCCCGCTC
DIAPS1R2	TTAGCTGGGGCYGACYTGRCTGGAGGACAC
DIAPS2F1	CAACATCACRRCSCTSAGCCCBTTCCTGGT
DIAPS2F2	CAGGAAGCCCADSACCATSACYACYACCAT
DIAPS2R1	CTGCAAGATAGAGGGNTTYDCBGCMACGCT
DIAPS2R2	AAGAATTTTABGCBGGGGMSACBTGGCTGG
DIAPR2F1	ATCAACATCCTCACCYTVYTKGTSACCTTC
DIAPR2F2	CAAGGAGGAATCCMADCACCATSARRATCA
DIAPR2R1	CTTCTCTGCCACTCAYGCCWTRWTRGGCAT
DIAPR2R2	CACTTGGCTGGAAGARAYRGAVGAKACCTC
DIAPR1F1	GTCAAAATTTCTAYRTBCCCWTKTCCAACA
DIAPR1F2	AATAGGATGCWRCWTYGARGGCTTCTTTGC
DIAPR1R1	ACAGTGCAGACAAGRYKYCCRTAGCAGAAG
DIAPR1R2	ATTCTTTCCACARCARAGRGTBRTGATCAT

Journal of Experimental Biology • Supplementary information

Table S2. Sampling protocol for assessing the density of neurons in different regions of the retina.

Cell population	Sampling protocol	No. of retinae sampled	Counting grid (µm)	Counting frame (µm)	Number of sites sampled	CE[1]
All photoreceptors	Main	3	800 X 800	35 X35	230-250	0.02-0.03
	Subsample	3	800 X 800	35 X35	20-30	0.03-0.05
Single cones	Main	3	800 X 800	35 X35	230-250	0.02-0.03
	Subsample	3	800 X 800	35 X35	20-30	0.03-0.05
Double cones	Main	3	800 X 800	35 X35	230-250	0.03-0.08
	Subsample	3	800 X 800	35 X35	20-30	0.06-0.09
RH1-expressing photoreceptors	Main	3	850 X 850	100 X 100	225-250	0.03-0.04
	Subsample	3	300 X 300	100 X 100	30-35	0.03-0.05
SWS1-expressing photoreceptors	Main	3	850 X 850	100 X 100	190-230	0.03-0.04
	Subsample	3	400 X 400	100 X 100	12-20	0.04-0.09

[1] Schaeffer's Coefficient of Error (CE) associated with each sampling protocol (Glaser and Wilson, 1998, Slomianka and West, 2005).
